# Effects of Vildagliptin, a Dipeptidyl Peptidase-4 Inhibitor, on the Parameters of Glucose Metabolism and the Cardio-Ankle Vascular Index in Individuals with Type 2 Diabetes

**DOI:** 10.3390/jcm13020481

**Published:** 2024-01-15

**Authors:** Daiji Nagayama, Hidetoshi Kawana, Yasuhiro Watanabe, Osamu Horikawa, Masahiro Ohira, Atsuhito Saiki

**Affiliations:** 1 Department of Internal Medicine, Nagayama Clinic, Oyama 323-0032, Tochigi, Japan; 2 Center of Diabetes, Endocrinology and Metabolism, Toho University, Sakura Medical Center, Sakura 285-0841, Chiba, Japan; yasuhiro.watanabe@med.toho-u.ac.jp (Y.W.); osamu.horikawa@med.toho-u.ac.jp (O.H.); atsuhito156@sakura.med.toho-u.ac.jp (A.S.); 3 Department of Diabetes and Metabolism, Chiba Kaihin Municipal Hospital, Chiba 261-0012, Chiba, Japan; h-kawana@umin.ac.jp; 4 Division of Diabetes, Metabolism and Endocrinology, Toho University Ohashi Medical Center, Meguro 153-8515, Tokyo, Japan; 600137om@sakura.med.toho-u.ac.jp

**Keywords:** vildagliptin, dipeptidyl peptidase-4 inhibitor, cardio-ankle vascular index, glycemic variability

## Abstract

DPP-4 inhibitors are frequently used as first-line agents for the treatment of type 2 diabetes in Japan. This study aimed to examine the effects of vildagliptin on glucose metabolism and arterial stiffness. Twenty treatment-naïve patients with type 2 diabetes (8 males and 12 females) received vildagliptin 50 mg twice daily for 6 months. Self-monitored blood glucose measurements and a 75 g OGTT were performed. Arterial stiffness was assessed using the CAVI. After the vildagliptin treatment, a significant decrease in the median HbA1c (from 8.3 to 6.4%) and fasting HOMA-β (from 26.1 to 34.5%), and a marginally significant decrease in the CAVI (from 8.9 to 8.4, *p* = 0.087) were observed. The glycemic variability parameters also improved, whereas the insulin sensitivity and oxidative stress remained unchanged. Participants with a lower glycemic variability on the 75 g OGTT after vildagliptin treatment showed a significant decrease in their CAVI. The baseline BMI was significantly higher for the participants with a decreased CAVI than in those with no change in their CAVI (24.5 vs. 20.8 kg/m^2^). After vildagliptin treatment, a decrease in the CAVI was observed, especially in the individuals with improved glycemic variability on the 75 g OGTT. Vildagliptin may be suitable for vascular protection in individuals with high glycemic variability and/or an elevated BMI.

## 1. Introduction

Even today, there remains a consensus that biguanides are the first-line treatment for type 2 diabetes [1]. However, of the currently available oral diabetic agents, the dipeptidyl peptidase-4 (DPP-4) inhibitors that were launched in 2009 are the most commonly used first-line agents in Japan [2]. The main reasons for the frequent use of DPP-4 inhibitors are their high safety profile and small number of nonresponsive cases. Although DPP-4 inhibitor monotherapy is expected to improve outcomes, its efficacy at preventing cardiovascular disease (CVD) remains controversial. For example, there is concern that DPP-4 inhibitors may promote adrenergically mediated cardiotoxicity, leading to heart failure events [3]. On the other hand, there are also conflicting reports that DPP-4 inhibitors contribute to improved long-term cardiac outcomes in individuals with heart failure with a preserved ejection fraction [4]. Ultimately, most DPP-4 inhibitors are recognized as having a neutral effect on vascular complications [5]. 

Vascular dysfunction manifesting as systemic arterial stiffening appears with aging, and is also associated with traditional CVD risk factors, including glucose intolerance [6,7]. The cardio-ankle vascular index (CAVI), an arterial stiffness parameter, has been developed to assess the stiffness of the arterial tree from the aortic origin to the ankle independent of blood pressure (BP) [8]. This parameter is known to be high in individuals with various atherosclerotic diseases, including coronary artery disease [9], cerebral infarction [10], carotid sclerosis [11], and chronic kidney disease [12]. In addition, a positive relationship between the CAVI and CVD risk factors has been reported [13]. Glucose intolerance may also contribute to systemic arterial stiffening, and a higher CAVI is generally observed in individuals with diabetes than in those without [14]. Since therapeutic interventions to reduce the CAVI may contribute to the prevention of future cardiovascular events [13], there is a need to clarify the effect of diabetes treatment on vascular dysfunction. 

Sitagliptin is the most widely used DPP-4 inhibitor in Japan and worldwide [15]. On the other hand, vildagliptin 50 mg twice daily has been reported to strongly reduce the 24 h mean plasma glucose (PG) levels compared to sitagliptin 50 mg once daily [16]. This may be because vildagliptin exerts a higher inhibitory effect on DPP-4 activity than sitagliptin [17]. The significance of vildagliptin monotherapy has been reviewed [18], and vildagliptin has even been shown to exert a stabilization effect on coronary plaques [19]. Furthermore, the additional efficacy of vildagliptin has also been reported in individuals with type 2 diabetes which is inadequately controlled by sulfonylureas [20] or biguanides [21]. On the other hand, the effect of vildagliptin on the CAVI has been not clarified.

Therefore, the present study aimed to determine the effects of vildagliptin on glucose metabolism and the CAVI in treatment-naïve patients with type 2 diabetes. Furthermore, the factors influencing changes in the CAVI were analyzed.

## 2. Materials and Methods

### 2.1. Subjects and Design

Twenty Japanese outpatients with type 2 diabetes mellitus were enrolled at the Center of Diabetes, Endocrinology, and Metabolism, Toho University, Sakura Medical Center, Sakura-city in Chiba, Japan. Type 2 diabetes mellitus was diagnosed based on the World Health Organization (WHO in Geneva, Switzerland) and American Diabetes Association (ADA in Arlington, Virginia, USA) criteria. The participants were counseled immediately after they were first diagnosed with diabetes, and provided guidance on diet and exercise, resulting in adequate diabetes control for more than three months. Individuals with a history of diabetes, severe diabetic retinopathy, severe hepatic dysfunction, CVD, and/or peripheral arterial disease were excluded from this study. Additionally, individuals with renal dysfunction equivalent to chronic kidney disease category 3a or higher [22] were also excluded because they might be unable to receive vildagliptin at the prescribed dose. The participants took vildagliptin 50 mg twice daily for 6 months. No other diabetic agents were administered during this study. 

### 2.2. Data Collection

All parameters were assessed using standardized methods. Height and body weight (BW) were measured, and body mass index (BMI) was calculated as follows: BW (kg) divided by height squared (m). Current smoking and habitual alcohol consumption status were determined using a questionnaire. Habitual alcohol consumption was defined as daily alcohol consumption.

Blood samples were collected from the antecubital vein in the morning after a 12 h fast to measure fasting plasma glucose (FPG, mg/dL), total cholesterol (TC, mg/dl), triglycerides (TG, mg/dL), high-density lipoprotein cholesterol (HDL-C, mg/dL), aspartate aminotransferase (AST, IU/L), alanine aminotransferase (ALT, IU/L), γ-glutamyl transpeptidase (γ-GTP, IU/L), and creatinine (mg/dL), using standardized methods. The urine albumin–creatinine ratio (UACR) was measured using turbidimetric immunoassay using a Superior-Microalbumin kit (Mitsubishi Chemistry Medicine, Tokyo, Japan). Low-density lipoprotein cholesterol (LDL-C) (mg/dL) was calculated using the Friedewald formula: LDL-C = (TC) − (HDL-C) − (TG/5). Estimated glomerular filtration rate (eGFR) was calculated using the following equation from the Japanese Society of Nephrology [23]: eGFR (mL/min/1.73 m^2^) = 194 × creatinine^−1.094^ × age^−0.287^ (× 0.739 if female).

### 2.3. Assessment of Glucose Metabolism Parameters

Serum biomarkers, including glycated hemoglobin (HbA1c, %), 1,5-anhydroglucitol (1,5 AG, µg/mL), and immunoreactive insulin (IRI, µg/mL), were assessed at baseline and after 6 months of vildagliptin treatment. The homeostatic model assessment for insulin resistance (HOMA-IR) and β-cell function (HOMA-β) were estimated using the following formulas [24]:

HOMA-IR = (FPG × IRI)/405

HOMA-β = (IRI × 360)/(FPG–63)

After enrollment, 6-point self-monitoring blood glucose (SMBG) measurements (before and 2 h after each of the three meals per day) were performed using the Medisafe FIT^®^ (Terumo Corporation, Tokyo, Japan) for two consecutive days at baseline (before vildagliptin treatment), and after 3 months and 6 months of vildagliptin treatment. The accuracy of the Medisafe FIT^®^ has been reported, with a coefficient of variation (CV) of less than 5%, and a measurable range of 20–600 mg/dL. However, some participants missed some SMBG measurements; therefore, data for 15 of the 20 participants were analyzed. The M-value, an index of glycemic variability, was estimated as the average of the M × (SMBG/SMBG) values and M × (SMBG/SMBG) = 10 × |log (BG/120)|^3^ [25].

The 75 g oral glucose tolerance test (OGTT) was conducted after overnight fasting at baseline and after 6 months of vildagliptin treatment. Blood samples were collected 0, 30, 60, and 120 min after glucose loading. As a measure of early insulin response to glucose, the insulinogenic index was calculated by dividing the increase in IRI from 0 to 30 min after glucose loading (ΔIRI 30) by the increase in PG from 0 to 30 min (ΔPG 30) as follows: [30-min IRI − fasting IRI (μIU/mL)]/[30-min PG − FPG (mg/dL)] [26]. To further investigate the effect of glycemic variability on arterial stiffness, participants were dichotomized according to ΔPG at 120 min of 75 g OGTT after 6 months of vildagliptin treatment, and ΔCAVI was compared between the two groups.

### 2.4. Measurement of Diacron-Reactive Oxygen Metabolites as a Parameter of Oxidative Stress

Serum samples were evaluated for oxidative stress using a diacron-reactive oxygen metabolite (d-ROM) test. d-ROM levels were measured using a kinetic spectrophotometric method (F.R.E.E System; Diacron, Italy), with an intra-assay CV of 2.1% and an inter-assay CV of 3.1%. Briefly, a serum sample (25 µL) was mixed with an acetic acid-buffered solution (pH 4.8) using a pipette to stabilize the hydrogen ion concentration, and a chromogenic substrate was added to the mixture. In an acidified medium, bivalent and trivalent iron from the protein component of the blood ionizes and acts as a catalyst to break down the hydroperoxide groups into alkoxy and peroxy radicals to form free radicals. The mixture was then incubated in the thermostatic block of the system and transferred to a cuvette containing colorless chromogen. The chromogen was oxidized by free radicals to radical cations with a magenta color, which was measured photometrically (505 nm) after centrifugation for 1 min. The intensity of the magenta color reflects the concentration of hydroperoxides in blood, which is proportional to the quantity of ROMs. The data were expressed in U. Carr (1 U. Carr corresponds to 0.08 mg/dL H_2_O_2_) [27].

### 2.5. Measurement of Lipoprotein Lipase Mass as a Parameter of Insulin Sensitivity

Lipoprotein lipase (LPL), which is presumably anchored to the surface of endothelial cells and hydrolyzes TG in the blood, is mainly produced in adipose and skeletal muscle tissues through the action of insulin. A sensitive immunoassay system using a specific monoclonal antibody against LPL can detect LPL mass in serum without heparin injection [28]. The preheparin serum level of the LPL mass (abbreviated as LPL mass hereafter) correlates negatively with the intra-abdominal visceral fat area evaluated using computed tomography [29] and with the severity of metabolic syndrome [30], suggesting that low LPL mass reflects insulin resistance.

LPL mass was measured using a sandwich enzyme-linked immunosorbent assay using a specific monoclonal antibody against bovine milk LPL, as described by Kobayashi et al. [28]. A commercial kit from Daiichi Pure Chemicals (Tokyo, Japan) was used in this study. For this assay system, linearity was observed from 5 to 400 ng/mL, with a within-run CV of 2.8% and between-day CV of 4.3%. However, the LPL mass could not be measured for one participant, and data for 19 of the 20 participants were analyzed. 

### 2.6. Measurements of the CAVI and Blood Pressure

Arterial stiffness was assessed by measuring CAVI using VaSera VS-1500 (Fukuda Denshi Co. Ltd., Tokyo, Japan). Measurements were started after a 5 min rest period. The pressure at all cuffs was maintained at 50 mm Hg to minimize the effect of cuff pressure on hemodynamics. CAVI was calculated using the following formula [8]: CAVI = a{2ρ × ln(Ps/Pd)/ΔP × PWV^2^} + b, where Ps is systolic blood pressure (SBP), Pd is diastolic blood pressure (DBP), ΔP is P − Pd, ρ is the blood density, PWV denotes the cardio-ankle pulse wave velocity, and a and b are constants.

### 2.7. Statistical Analysis

SPSS software (version 27.0.1, Chicago, IL, USA) was used for the statistical analyses. Most data are expressed as medians (interquartile range (IQR)) or percentages. Only changes in (Δ) PG on the 75 g OGTT are expressed as the mean ± standard deviation. The Friedman test, followed by the post hoc Bonferroni method, were performed to examine the differences in clinical variables between baseline, after 3 months, and after 6 months of vildagliptin treatment. The Wilcoxon signed-rank test was also performed for comparisons between two points: baseline and after 6 months of treatment. The Mann–Whitney U test or Fisher’s exact test was performed to examine differences in clinical variables between participants with and without a decrease in CAVI. In all comparisons, two-sided *p* values less than 0.05 were considered statistically significant. However, for *p* values in the range of 0.05 to 0.1, the term ‘marginally significant’ was used.

## 3. Results

### 3.1. Participant Characteristics at the Baseline and after Vildagliptin Treatment

The clinical profiles of this study’s participants are presented in Table 1. Twenty Japanese outpatients (eight males and 12 females; median age, 60 years) with type 2 diabetes were enrolled in this study. At the baseline, some participants had hypertension (20.0%) and/or dyslipidemia (20.0%). After 6 months of vildagliptin treatment, significant decreases in the FPG, HOMA-β, HbA1c, and γ-GTP, and an increase in the 1,5-AG were observed. The median CAVI showed a marginally significant decrease (from 8.9 to 8.4; *p* = 0.087). The BMI, BP, HOMA-IR, hepatic and renal parameters, d-ROMs, and LPL mass did not change significantly after 6 months of treatment.

Most participants did not show an elevated UACR and none had diabetic retinopathy. Hypoglycemia, defined as a PG level < 70 mg/dL, was not observed in any participant during the study period.

### 3.2. Diurnal Variation in Self-Monitoring Blood Glucose (SMBG) Levels

**Figure 1:**
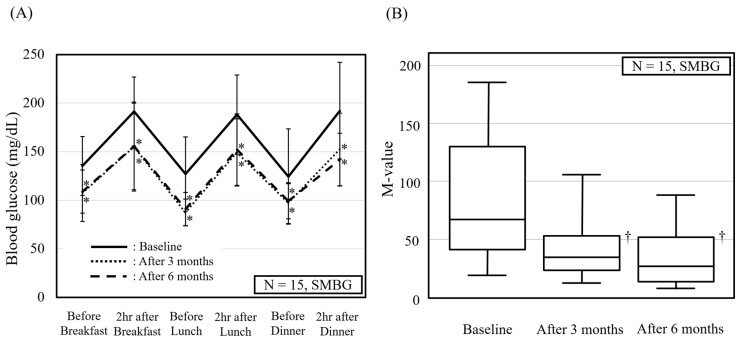
Diurnal variation in self-monitoring blood glucose (SMBG) measurements. (**A**) Summary of 6-point SMBG profiles at baseline and after 3 and 6 months of vildagliptin treatment. * *p* < 0.05 vs. baseline, analyzed using Wilcoxon signed-rank test. (**B**) Comparison of M-values estimated from SMBG measurements. Box and whisker plot demonstrating changes in M-value between baseline and after 3 and 6 months of vildagliptin treatment. Bottom of the box is 25th percentile and top of the box is 75th percentile. Central line is median. Vertical lines represent 10th and 90th percentiles. ^†^ *p* < 0.05 vs. baseline, analyzed using Friedman test followed by post hoc Bonferroni test.

The participants performed SMBG measurements six times a day for two consecutive days at the baseline and after three and 6 months of vildagliptin treatment. Figure 1A shows the daily blood glucose trends and Figure 1B presents the M-values. Some SMBG measurements were missing for some participants; therefore, the data for 15 of the 20 participants were analyzed.

The results show that vildagliptin treatment reduced both the pre- and postprandial blood glucose levels. There was no difference in the blood glucose levels between the 3- and 6-month vildagliptin treatment groups. Similarly, the M-values were significantly reduced after 3 and 6 months of vildagliptin treatment.

### 3.3. Oral Glucose Tolerance Test at the Baseline and after 6 Months of Vildagliptin Treatment

**Figure 2:**
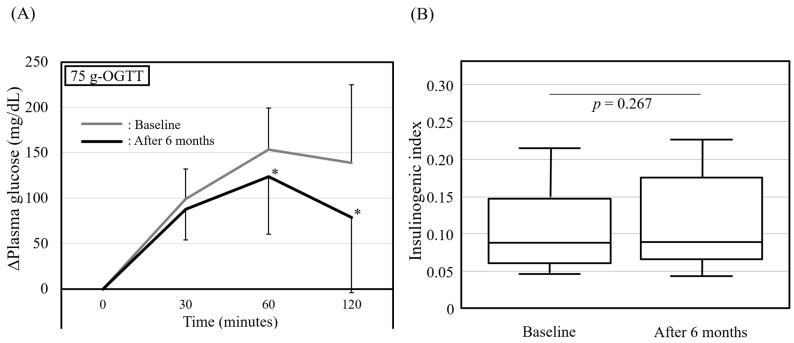
A 75 g OGTT conducted at baseline and after 6 months of vildagliptin treatment. (**A**) Changes in plasma glucose during OGTT are expressed as mean ± standard deviation. * *p* < 0.05 vs. baseline, analyzed using Wilcoxon signed-rank test. (**B**) Comparison of insulinogenic index under OGTT. Box and whisker plot demonstrating change in insulinogenic index between baseline and after 6 months of vildagliptin treatment. Bottom of the box is 25th percentile and top of the box is 75th percentile. Central line is median. Vertical lines represent 10th and 90th percentiles. OGTT: oral glucose tolerance test.

The results of the OGTTs performed with a 75 g glucose load were compared between the baseline and after 6 months of vildagliptin treatment, as shown in Figure 2. Elevations in the PG at 60 and 120 min after glucose loading were significantly suppressed after 6 months of vildagliptin treatment (Figure 2A), whereas the insulinogenic index showed no significant change (Figure 2B). In other words, 6 months of vildagliptin treatment did not affect the ΔPG or insulin secretion at 30 min after glucose loading, but prevented a glucose spike at 60–120 min.

### 3.4. Relationship of the CAVI with Glycemic Variability for the 75 g OGTT after 6 Months of Vildagliptin Treatment

**Figure 3:**
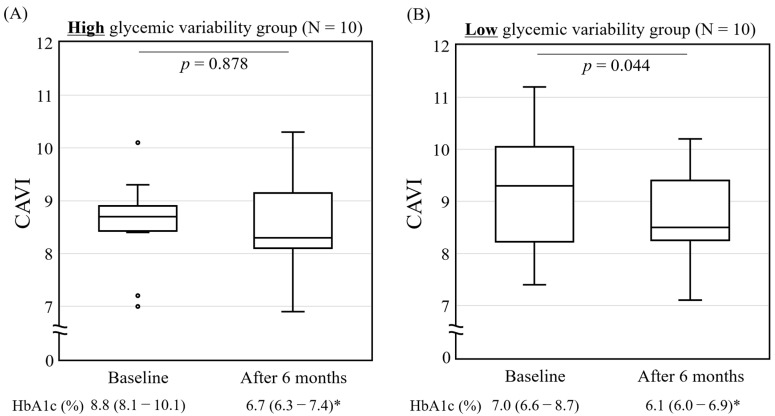
Relationship of CAVI with glycemic variability for the 75 g OGTT after 6 months of vildagliptin treatment. Changes in CAVI before and after 6 months of vildagliptin treatment in (**A**) higher glycemic variability group and (**B**) lower glycemic variability group. Higher/lower glycemic variability is defined as Δ plasma glucose at 120 min of 75 g OGTT >/≤ 50th percentile after 6 months of vildagliptin treatment. Box and whisker plot demonstrating change in insulinogenic index between baseline and 6 months after vildagliptin treatment. Bottom of the box is 25th percentile and top of the box is 75th percentile. Central line is median. Vertical lines represent 10th and 90th percentiles. * *p* < 0.01, analyzed using Wilcoxon signed-rank test. OGTT: oral glucose tolerance test; HbA1c: glycated hemoglobin; BMI: body mass index.

Next, participants were dichotomized using the ΔPG 120 min after glucose loading into a higher glycemic variability group (ΔPG: minimum 76, maximum 271, median 128 mg/dL) and a lower glycemic variability group (ΔPG: minimum −52, maximum 62, median 26 mg/dL). The CAVI was then compared between the two groups, as shown in Figure 3. The CAVI [median (IQR)] did not change significantly in the higher glycemic variability group [8.7 (8.4–8.9) to 8.3 (8.1–9.2), *p* = 0.878] (Figure 3A), but decreased significantly in the lower glycemic variability group [9.3 (8.2–10.1) to 8.5 (8.3–9.4), *p* = 0.044] (Figure 3B) after 6 months of vildagliptin treatment. Both groups showed a significant decrease in their HbA1c levels after 6 months of vildagliptin treatment.

### 3.5. Comparison of Participants with or without a CAVI Decrease after 6 Months of Vildagliptin Treatment

**Figure 4:**
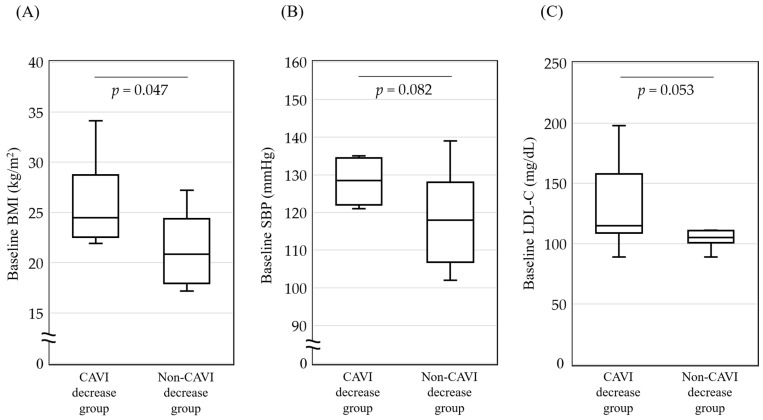
Comparison of baseline (**A**) BMI, (**B**) SBP, and (**C**) LDL-C in participants with or without decrease in CAVI after 6 months of vildagliptin treatment. Bottom of the box is 25th percentile and top of the box is 75th percentile. Central line is median. Vertical lines represent 10th and 90th percentiles. *P* values were analyzed using Mann–Whitney U test. BMI: body mass index; SBP: systolic blood pressure; LDL-C: low-density lipoprotein cholesterol; CAVI: cardio-ankle vascular index.

Finally, as shown in Table 2, all the participants were divided into two groups: those with a decrease in their CAVI (ΔCAVI < 0; CAVI decrease group, 60.0%), and those with no change or increase in their CAVI after 6 months of vildagliptin treatment (ΔCAVI ≥ 0; non-CAVI decrease group, 40.0%). The main characteristics of the two groups were compared. The CAVI decrease group had a higher baseline BMI than the non-CAVI decrease group (median, 24.5 vs. 20.8%; *p* = 0.047). There was also a trend towards a higher SBP and LDL-C in the CAVI decrease group than in the non-CAVI decrease group; however, the differences were not significant (Figure 4). There were no differences between the two groups in terms of the baseline values or changes in the glucose metabolism parameters.

## 4. Discussion

In the present study, vildagliptin treatment for 6 months reduced both the fasting and postprandial PG, and increased the HOMA-β, but did not change the HOMA-IR, LPL mass, or d-ROMs. These findings indicate that the PG-lowering effect of vildagliptin was mainly due to an improvement in β-cell function. Additionally, a marginally significant decrease in the CAVI was observed. When we examined the distribution of the CAVI using a subgroup analysis, the participants with relatively low glycemic variability on the 75 g OGTT after vildagliptin treatment showed a significant decrease in the CAVI, whereas those with relatively high variability did not differ. We also observed that the individuals with a decreased CAVI had a significantly higher baseline BMI. The strength of the present study lies in the detailed investigation of the effects of vildagliptin on arterial stiffness and glucose metabolism.

Several plausible mechanisms can be inferred to explain the vascular function-improving effects of vildagliptin. First, vildagliptin may decrease the CAVI by reducing glucotoxicity. Chronic hyperglycemia increases the advanced glycation end-products (AGEs), which induce inflammation, oxidative stress, calcium deposition in vessel walls, and the apoptosis of vascular smooth muscle, resulting in the development of organic arteriosclerotic lesions [31]. Therefore, the hypoglycemic effect of vildagliptin is expected to inhibit this atherosclerotic process. However, the group with a decreased CAVI did not necessarily show a greater improvement in their glucose metabolic parameters than the group without a decreased CAVI. This finding is consistent with our previous study showing that glibenclamide, a conventional sulfonylurea, does not affect the CAVI, even though it exerts an apparent hypoglycemic effect [32]. Similarly, the ACCORD [33] and ADVANCE [34] trials have reported that the intensive management of diabetes does not necessarily contribute to a reduction in cardiovascular events. On the other hand, therapeutic interventions focusing on postprandial hyperglycemia, rather than on chronic hyperglycemia, may improve vascular dysfunction. Our results reveal that a lower ΔPG on the 75 g OGTT was associated with a decreased CAVI. This finding is consistent with previous reports showing an association between an increased CAVI and postprandial hyperglycemia [35,36,37]. A glucose spike is known to induce endothelial dysfunction via activation of reactive oxygen species [38] and monocyte adhesion involved in vascular inflammation [39]. Given that an increased CAVI reflects endothelial dysfunction [40,41], we hypothesize that vildagliptin improves the endothelial function by suppressing glucose spikes. This hypothesis may be similar to the mechanism by which the alpha-glucosidase inhibitor decreases the CAVI [42]. On the other hand, since there were some participants who did not show a suppressed glucose spike or a decreased CAVI despite vildagliptin treatment, the reason for this difference in response should be investigated further.

In the present study, it seems contradictory that the M-value was not associated with the CAVI (Table 2), unlike the ΔPG on the 75 g OGTT. Indeed, the M-value is a glycemic variability index designed to reflect not only hyperglycemia, but also hypoglycemia. However, because DPP-4 inhibitor monotherapy does not induce hypoglycemia, the M-value may merely serve as an index of hyperglycemia, similar to the HbA1c. 

Next, the higher baseline BMI of the CAVI decrease group versus that of the non-CAVI decrease group may partially explain the pleiotropic vascular effects of vildagliptin. Considering that a high BMI primarily reflects increased body fat mass, the pharmacologic mechanisms possessed by DPP4 inhibitors may be reasonable for vascular protection. DPP-4 is a novel adipokine mainly produced by adipose tissue [43]. The serum concentration of DPP-4 correlates positively with the BMI, and its expression is linked to adipocyte size in visceral adipose tissue. Although unfortunately not included in the observations, the adoption of a visceral fat area or abdominal obesity indices may have helped to identify more clearly those individuals for whom DPP4 inhibitors improved vascular function. Furthermore, DPP-4 inhibition in experimental animal models has reduced inflammation and oxidative stress, and improved endothelial function [44]. In other words, DPP-4 may exert a vascular toxicity directly without the mediation of glucose intolerance. Therefore, in terms of vascular protection, it seems reasonable to administer DPP-4 inhibitors to individuals with obesity who express high levels of DPP-4. Furthermore, the CAVI decrease group showed a marginally significant higher baseline SBP and LDL-C, suggesting the beneficial vascular effect of DPP-4 inhibitors for individuals with relatively high SBP and LDL-C. The efficacy of DPP4-inhibitors on blood pressure and lipid profiles has attracted much attention [45], and further detailed mechanistic elucidation is desirable. 

Finally, another possibility is that the reduction in hepatic insulin resistance by vildagliptin contributed to the decreased CAVI. Tahara et al. [46] have already reported that anagliptin, a DPP-4 inhibitor, reduced the CAVI and improved liver dysfunction. Similarly, our results show that vildagliptin reduced the γ-GTP levels. Ectopic liver lipid has been reported to exacerbate hepatic insulin resistance, promote systemic inflammation, and increase the risk of developing both type 2 diabetes and CVD [47]. However, there was no significant difference in the Δγ-GTP between the CAVI decrease and non-CAVI decrease groups, and the d-ROMs did not change. Therefore, this hypothesis cannot be substantiated. 

In addition to improving liver function, vildagliptin has also been reported to improve renal inflammatory markers and, consequently, reduce proteinuria, independent of its PG-lowering effect [48]. Vildagliptin also provides clinically important reductions in glycated hemoglobin, without increasing weight or the risk of hypoglycemia, even in patients with severe chronic kidney disease [49]. Furthermore, DPP-4 inhibitors are known to reduce insulin resistance and oxidative stress [50,51,52]. However, neither the UACR nor the parameters of insulin sensitivity and oxidative stress changed after vildagliptin treatment in the present study. The participants in this study may not have been exposed to high levels of insulin resistance, oxidative stress, or renal toxicity at the time of enrollment. However, the CAVI may decrease, even by improving the pathophysiology of type 2 diabetes. We previously reported that the insulin-sensitizer pioglitazone decreases the CAVI and, concomitantly, increases adiponectin [53]. Glimepiride, a third-generation sulfonylurea, exerts a CAVI-decreasing effect by ameliorating insulin resistance and oxidative stress [32]. These findings suggest that the arterial stiffening observed in individuals with type 2 diabetes is associated with postprandial hyperglycemia, insulin resistance, and oxidative stress. Large-scale cohort studies focusing on type 2 diabetes with organ damage and/or insulin resistance are desirable to further clarify the effects of vildagliptin.

This study has some limitations, and further research is needed to address these issues. First, some participants had missing LPL mass or SMBG values, but they were not excluded from the analysis because of the small sample size of the present study. The small sample size is a limitation that should be considered when interpreting the results. Other potential confounders, such as medication adherence, lifestyle factors, and comorbidities that were not confirmed in this study, may have interfered with the results, and are recognized as limitations of this study. Next, although the SMBG was adopted in this study, continuous glucose monitoring would have provided a more detailed assessment of glycemic variability. Finally, to clarify whether vildagliptin improves vascular function through mechanisms other than the amelioration of glucotoxicity, it would be desirable to include a control group with comparable hypoglycemic control using an agent other than vildagliptin.

## 5. Conclusions

After 6 months of vildagliptin treatment, the parameters of glucose metabolism, including insulin secretion, improved. A decrease in the CAVI was also observed, especially in individuals with improved glycemic variability on the 75 g OGTT. DPP-4 inhibitors may be considered as the first-line treatment for individuals with type 2 diabetes when biguanides are difficult to use or when insulin secretion is impaired. In particular, vildagliptin may be suitable for vascular protection in individuals with high glycemic variability and/or an elevated BMI.

## Figures and Tables

**Table 1:** Participant characteristics at baseline and after vildagliptin treatment.

Variables	Baseline	After 3 Months	After 6 Months	*p* Value ^†^
N (male/female)	8/12	-	-	-
Age (years)	60 (54–65)	-	-	-
Smoking (%)	30.0	-	-	-
Habitual alcohol drinking (%)	50.0	-	-	-
BMI (kg/m^2^)	23.8 (21.9–25.7)	-	23.8 (21.3–25.8)	0.615
SBP (mmHg)	126 (121–133)	-	125 (115–137)	0.472
DBP (mmHg)	78 (75–88)	-	77 (72–81)	0.856
CAVI	8.9 (8.3–9.5)	-	8.4 (8.1–9.3)	0.087
FPG (mg/dL)	152 (129–180)	115 (107–148) *	137 (123–148) *	0.044
Fasting IRI (μU/mL)	5.7 (3.8–9.2)	-	5.6 (3.8–8.7)	0.845
HOMA-IR	2.1 (1.4–3.7)	-	2.3 (1.2–3.2)	0.184
HOMA-β (%)	26.1 (10.5–36.0)	-	34.5 (15.7–39.7)	0.013
HbA1c (%)	8.3 (6.8–9.5)	6.7 (6.2–7.2) *	6.4 (6.1–7.1) *	< 0.001
1,5-AG (µg/mL)	5.4 (3.1–9.9)	-	11.7 (7.8–15.2)	< 0.001
LDL-C (mg/dL)	111 (103–127)	115 (88–137)	119 (94–129)	0.520
HDL-C (mg/dL)	56 (40–62)	55 (46–61)	54 (44–66)	0.962
TG (mg/dL)	96 (69–118)	120 (63–212)	90 (53–147)	0.006
AST (IU/L)	20 (16–23)	19 (17–23)	21 (15–25)	0.383
ALT (IU/L)	20 (15–25)	17 (14–20)	18 (15–20)	0.149
γ-GTP (IU/L)	25 (19–30)	19 (15–27) *	19 (16–27) *	<0.001
Creatinine (mg/dL)	0.60 (0.54–0.70)	0.66 (0.53–0.71)	0.66 (0.51–0.68)	0.815
eGFR (mL/min/1.73 m^2^)	87.3 (77.1–100.9)	88.6 (71.9–97.5)	91.5 (74.4–96.5)	0.815
UACR (mg/g·Cr)	14.3 (8.9–17.7)	13.3 (9.4–19.2)	13.3 (9.4–19.7)	1.000
d-ROMs (U. Carr)	387 (344–424)	-	371 (346–397)	0.421
LPL mass (ng/mL) (N = 19)	63.1 (46.9–71.1)	-	60.5 (51.6–79.5)	0.296
Hypertension (%)	20.0	-	-	-
Dyslipidemia (%)	20.0	-	-	-

Data are expressed as medians (IQR) or percentages. ^†^ Wilcoxon signed-rank test for comparison between baseline and 6 months; Friedman test for comparison among baseline, 3 months, and 6 months. * *p* < 0.05 vs. baseline estimated using post hoc Bonferroni test. BMI: body mass index; SBP: systolic blood pressure; DBP: diastolic blood pressure; CAVI: cardio-ankle vascular index; FPG: fasting plasma glucose; IRI: immunoreactive insulin; HOMA-IR: homeostatic model assessment of insulin resistance; HOMA-β: homeostatic model assessment of β-cell function; HbA1c: glycated hemoglobin; 1,5-AG: 1,5-anhydroglucitol; LDL-C: low-density lipoprotein cholesterol; HDL-C: high-density lipoprotein cholesterol; TG: triglyceride; AST: aspartate aminotransferase; ALT: alanine aminotransferase; γ-GTP: γ-glutamyl transpeptidase; eGFR: estimated glomerular filtration rate; UACR: urine albumin–creatinine ratio; d-ROMs: diacron-reactive oxygen metabolites; LPL: lipoprotein lipase; IQR: interquartile range.

**Table 2:** Comparison of characteristics of participants with or without decrease in CAVI after 6 months of vildagliptin treatment.

Variables	CAVI Decrease Group	Non-CAVI Decrease Group	*p* Value *
N (male/female)	12 (4/8)	8 (4/4)	0.648
Age (years)	60.0 (49.0–64.0)	60.5 (59.5–65.0)	0.510
BMI (kg/m^2^)	24.5 (23.1–26.8)	20.8 (18.6–24.3)	0.047
SBP (mmHg)	129 (124–134)	118 (108–128)	0.082
DBP (mmHg)	79 (75–90)	77 (71–80)	0.176
CAVI	8.9 (8.3–9.6)	9.1 (8.3–9.4)	1.000
FPG (mg/dL)	148 (128–187)	153 (133–168)	0.671
ΔFPG (mg/dL)	−7 (−91–4)	−14 (−35–−4)	0.246
Fasting IRI (μU/mL)	6.8 (4.4–9.2)	4.8 (2.4–7.5)	0.463
HOMA-IR	2.8 (1.8–3.7)	1.5 (0.90–2.7)	0.305
ΔHOMA-IR	0 (−3.7–3.4)	−0.4 (−1.0–1.6)	0.537
HOMA-β (%)	25.0 (11.8–36.6)	31.5 (7.7–36.5)	0.847
ΔHOMA-β (%)	4.6 (−8.2–55.5)	2.2 (−3.2–26.7)	0.440
Insulinogenic index	0.09 (0.06–0.12)	0.08 (0.06–0.15)	0.968
M-value	16.4 (7.6–24.6)	8.8 (6.7–24.3)	0.678
ΔM-value	−4.9 (−16.8–−0.5)	−7.8 (−15.0–−4.1)	0.763
HbA1c (%)	7.3 (6.7–9.4)	9.6 (8.7–12.1)	0.521
ΔHbA1c (%)	−0.7 (−6.5–−0.1)	−2.0 (−4.9–0.0)	0.334
1,5-AG (µg/mL)	6.5 (3.6–11.6)	4.3 (2.7–6.8)	0.384
Δ1,5-AG (µg/mL)	3.7 (2.2–5.3)	3.1 (0.5–5.6)	0.643
LDL-C (mg/dL)	115 (110–157)	105 (102–110)	0.053
HDL-C (mg/dL)	57 (38–62)	51 (46–78)	0.817
TG (mg/dL)	94 (77–114)	107 (68–127)	0.787
AST (IU/L)	20 (17–22)	18 (14–23)	0.460
ALT (IU/L)	21 (19–25)	16 (15–23)	0.314
γ-GTP (IU/L)	25 (20–32)	23 (18–30)	0.817
Δγ-GTP (IU/L)	−5 (−25, 11)	−3 (−25, 2)	0.613
Creatinine (mg/dL)	0.60 (0.55–0.64)	0.61 (0.51–0.77)	1.000
eGFR (mL/min/1.73 m^2^)	85.6 (71.8–100.8)	92.8 (80.0–101.3)	0.910
UACR (mg/g·Cr)	14.3 (7.1–18.6)	13.8 (10.1–17.7)	0.792
d-ROMs (U. Carr)	395 (368–436)	347 (304–408)	0.270
LPL mass (ng/mL) (N = 19)	67.7 (60.3–71.0)	49.4 (41.1–63.4)	0.384

Data are expressed as medians (IQR) or percentages. * Mann–Whitney U test was used to compare baseline characteristics between two groups. Δ denotes the change in each variable during 6 months of treatment. BMI: body mass index; SBP: systolic blood pressure; DBP: diastolic blood pressure; CAVI: cardio-ankle vascular index; FPG: fasting plasma glucose; IRI: immunoreactive insulin; HOMA-IR: homeostatic model assessment of insulin resistance; HOMA-β: homeostatic model assessment of β-cell function; HbA1c: glycated hemoglobin; 1,5-AG: 1,5-anhydroglucitol; LDL-C: low-density lipoprotein cholesterol; HDL-C: high-density lipoprotein cholesterol; TG: triglyceride; AST: aspartate aminotransferase; ALT: alanine aminotransferase; γ-GTP: γ-glutamyl transpeptidase; eGFR: estimated glomerular filtration rate; UACR: urine albumin–creatinine ratio; d-ROM: diacron reactive oxygen metabolites; LPL: lipoprotein lipase mass; IQR: interquartile range.

## Data Availability

The data that support the findings of this study are not publicly available because they contain information that could compromise the privacy of the research participants. Further inquiries may be directed to the corresponding author.
